# The Role of Probiotics *Limosilactobacillus reuteri*, *Ligilactobacillus salivarius*, and *Lactobacillus johnsonii* in Inhibziting Pathogens, Maintaining Gut Health, and Improving Disease Outcomes

**DOI:** 10.3390/ijms27031545

**Published:** 2026-02-04

**Authors:** Li Li, Xiangqi Qiu, Shengyong Lu, Haitao Yu, Panpan Lu, Sumei Zeng, Aihua Deng, Min Zhu, E Xu, Jin Niu

**Affiliations:** 1 Key Laboratory of Animal Genetics, Breeding and Reproduction in the Plateau Mountainous Region, Ministry of Education, College of Animal Science, Guizhou University, Guiyang 550025, China; 2 State Key Laboratory of Animal Nutrition and Feeding, College of Animal Science and Technology, China Agricultural University, Beijing 100193, China; 3 Guangdong Provincial Key Laboratory of Animal Nutrition Control, College of Animal Science, South China Agricultural University, Guangzhou 510642, China; 4 School of Medical Humanities, China Medical University, Shenyang 110122, China

**Keywords:** research advances, probiotics, inhibit pathogen, intestinal health, disease outcomes

## Abstract

As the critical component of the gastrointestinal tract, which lives in trillions of gut microorganisms, in a healthy state, the host interacts with the gut microbiota and is symbiotic. The species *Limosilactobacillus reuteri*, *Ligilactobacillus salivarius*, and *Lactobacillus johnsonii* are indigenous gut commensal bacteria that are mainly found in the digestive tracts. These three bacteria possess a variety of characteristics that reflect their ability to adapt to the gastrointestinal environment. Herein, we summarize the current progress of research on the probiotic properties of these strains in terms of their ability to protect against harmful pathogens, maintain intestinal health, and improve disease outcomes. These bacteria can impact the intestinal barrier function and enhance intestinal immunity through various mechanisms, such as upregulating the tight-junction protein expression and mucin secretion of intestinal epithelial cells, adjusting and balancing the gut microbiota, and blocking pro-inflammatory cytokine production. They have been shown to ameliorate intestinal inflammation in animal models and provide protective effects against various healthy issues in humans, including diarrhea, constipation, colorectal cancer, obesity, and liver diseases. However, the detailed mechanisms of certain strains remain unclear.

## 1. Introduction

The gut microbiota is a crucial component of the gastrointestinal tract and plays a key role in maintaining host physiological functions. Many studies indicate that certain diseases in animals and humans, such as inflammatory bowel disease (IBD), obesity, metabolic syndrome, autism, and some cancers, are closely associated with impaired gut microbiota integrity [1,2]. Probiotics are defined as “live microorganisms which when intake in adequate amounts can benefit on host health” by the FAO/WHO [3]. Recently, probiotics have become increasingly frequent in the diet due to their health benefits. Notably, *Limosilactobacillus reuteri* (formerly named *Lactobacillus reuteri*, *L. reuteri*), *Ligilactobacillus salivarius* (formerly named Lactobacillus salivarius, *L. salivarius*), and *Lactobacillus johnsonii* (*L. johnsonii*) are widely used in human and animal production because of their beneficial properties [4]. *L. reuteri* can produce antimicrobial molecules, such as organic acids, ethanol, and reuterin [5]. *L. johnsonii* has many metabolites, including short-chain fatty acids, bacteriocins, and hydrogen peroxide [6]. *L. salivarius* is a well-characterized bacteriocin producer and probiotic organism, many of which are producers of unmodified bacteriocins of sub-classes IIa, IIb, and IId [7]. *L. salivarius* also exhibits tolerance to acid and bile salts, thus allowing for a higher survival rate in the gastrointestinal tract (GIT) [8].

*L. reuteri* has multiple probiotic effects on humans and animals, including intestinal colonization, host immune system regulation, broad-spectrum antimicrobial compound production and secretion, and prevention of diarrhea and colitis. Furthermore, *L. reuteri* has been used to study the intestinal commensal bacteria ecology and evolution model in vertebrates [9]. *L. johnsonii* has been used as a probiotic to treat diseases, showing specific advantages in the treatment of a variety of diseases, and can guarantee the health of production and daily life [10]. *L. salivarius* enhances immune function, inhibits pathogen colonization, increases animal production, and can also be used to treat chronic diseases [11]. They play an important role as probiotics and have great application prospects in biomedicine and livestock production.

In the present review, we summarize and discuss the current progress of research on the probiotic properties of these strains in terms of their ability to protect against harmful pathogens and maintain intestinal health, and their function in disease improvement, which hopefully will provide a reference for the effective application of these strains in the clinical and nutritional fields. 

## 2. Inhibits Pathogens and Ameliorates Damage

Pathogen infections are a serious and basic category of diarrhea, inflammation, and other organ damage caused by pathogen invasion. These pathogens that cause infections are mainly viruses and bacteria [12]. One of the most important characteristics of probiotics is that they can inhibit pathogenic microorganisms and bring beneficial effects to the host [13]. Herein, we summarize the effects of different strains of *L. reuteri*, *L. salivarius*, and *L. johnsonii* on the main pathogenic factors and their possible mechanisms (Table 1).

From Table 1, we can see that these strains were isolated from different hosts, including humans, pigs, chickens, calves, and lambs. Based on the available information, we could find *L. reuteri*, *L. salivarius*, and *L. johnsonii* inhibit both viruses and bacteria; they all inhibit *E. coli*, *Salmonella*, *H. pylori*, and *C. jejuni*. What is special is that *L. reuteri* had the ability to protect *P. gingivalis*, *E. faecalis*, *F. verticillioides*, *C. albicans*, *Shigella sonne*, *Shigella flexneri*, *Vibrio cholerae*, PCV2, and Influenza A/PR8, LD50 infection. *L. johnsonii* inhibited WSSV, *Endogenous pathogenic bacteria*, RSV, and *Clostridium perfringens*, especially for *L. salivarius*, which can protect *Aspergillus hydrophilus*, *Cronobacter sakazakii,* Infectious, IBDV, Uremic toxins, *Mycotoxin*, and HIV-1 infection. The mechanisms by which *L. reuteri*, *L. salivarius*, and *L. johnsonii* inhibit pathogens are summarized in Table 1. We can observe that they fight against pathogen infection, mainly through producing antimicrobial molecules, regulating the intestinal microbiota, reducing the inflammatory response, and enhancing host defense.

## 3. Maintains Intestinal Health and Homeostasis

The gut is the largest immune organ and is involved mainly in digestion, absorption, immune homeostasis, endocrine regulation, and other physiological functions, and its health is a direct response to the characteristics of the organism [113]. Gut autochthonous commensal *L. reuteri*, *L. salivarius*, and *L. johnsonii* are utilized mainly as food or food supplements, and the gastrointestinal tract is an important part of their function. Understanding their effects on the intestinal tract allows us to visualize their functions.

### 3.1. Improves the Intestinal Barrier

**Figure 1:**
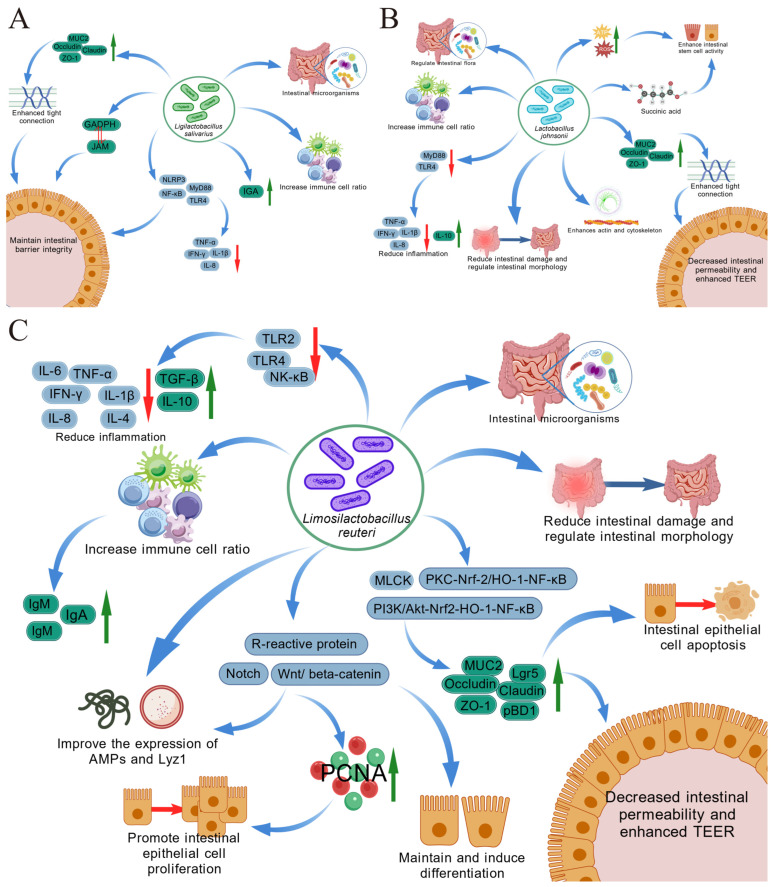
Different mechanisms underlying the effects of *L. salivarius* strains (**A**), *L. johnsonii* strains (**B**), and *L. reuteri* strains (**C**) on the intestinal barrier. Created with BioGDP.com [114].

The intestinal barrier is an important component of intestinal immunity, and *L. salivarius*, *L. johnsonii*, and *L. reuteri* can modulate intestinal immune responses by affecting the intestinal barrier. The intestinal barrier consists of four barriers, including mechanical, chemical, microbial, and immune barriers. Different mechanisms underlying the impacts of *L. salivarius*, *L. johnsonii*, and *L. reuteri* strains on the intestinal barrier is demonstrated in Figure 1.

Firstly, *L. salivarius*, *L. johnsonii*, and *L. reuteri* strains improve the intestinal barrier by impacting the tight-junction protein expression. For example, *L. salivarius* YL20 has been demonstrated to inhibit the invasion of *Chlamydia* sakazaki infection in the HT-29 and Caco-2 monolayer cell model, increasing ZO-1 and occludin expression. This strain also enhances intestinal barrier function by increasing the goblet cell count, MUC-2 levels, and ZO-1 expression in mouse intestinal organoids [87]. *L. salivarius* LI01 can effectively restore the intestinal barrier biomarkers Claudin-1 and MUC2 expression [115]. *L. salivarius* CPU-01 upregulated the colonic ZO-1 and Occludin expressions [116]. *L. salivarius* CML352 significantly increased Muc-2 to improve the intestinal barrier [117]. *L. salivarius* SNK-6 has been shown to upregulate the jejunum intestinal barrier-related mRNAs ZO-1, CLDN1, and MUC2 expressions [118]. About *L. johnsonii*, the results showed that *L. johnsonii* strain MG enhanced the intestinal barrier integrity through the interaction between GAPDH and the junctional adhesion molecule-2 (JAM-2), which showed that GAPDH interacts and docks with JAM-2 by the two peptides, 11GRIGRLAF18 at the N-terminus, and 323SFTCQMVRTLLKFATL338 at the C-terminus [119,120]. *L. johnsonii* MG pretreatment enhanced intestinal barrier function, protected against *Enterococcus faecium*-induced damage, and increased ZO-1 expression of Caco-2 cells [121]. Treatment with *L. johnsonii* N5 increased the expression levels of ZO-1, closure protein, and cytoprotective HSP70 under physiological conditions, and it alleviated colitis [122]. *L. johnsonii* L531 alleviated *Salmonella typhimurium*-induced damage through activating tight-junction protein expression and inhibiting the TLR4/NF-κB/NLRP3 signaling pathway [123]. *L. reuteri* strains LR1, DSM 17938, and 1563F had a protective effect against ETEC-induced damage to the mucosa, mainly through increased expression of ZO-1 [124,125,126]. *L. reuteri* 2892 upregulated the expression of tightly connected molecules ZO-1 and claudin-4 and inhibited the expression of metalloproteinase (MMP-2) and MMP-9 [127]. Supplementation with *L. reuteri* 81 significantly increased the gene expression of the ileum tight-junction protein *ZO-1* [128]. Administration of *L. reuteri* 4569 improved the expression of barrier-protective tight-junction protein (TJ) and cell-protective heat shock protein (HSP) 70 and HSP25 [129]. *L. reuteri B1/1* was even able to increase those of the tight-junction-related genes *CLDN1* and *OCLN* [130]. In this sense, several studies have found that different strains of *L. salivarius*, *L. johnsonii*, and *L. reuteri* improved tight-junction protein, thereby enhancing intestinal barrier function.

Secondly, *L. salivarius*, *L. johnsonii*, and *L. reuteri* strains can also improve the intestinal barrier through regulating the intestinal flora. It has been reported that *L. salivarius* CPU-01 and LS160 regulated the intestinal flora, maintained the intestinal structure, and enhanced intestinal barrier function [116,131]. *L. salivarius* LI01, CML352, and SNK-6 significantly altered the Firmicutes/Bacteroides ratio to protect the intestinal barrier structure [117,118,132]. *L. Salivarius* PS21603 reduced *Escherichia*, and increased *Bifidobacterium* to improve the intestinal barrier in piglets [133]. A consortium of *L. salivarius* 7247 and *L. curvatureus* 2029 strains prevented intestinal barrier dysfunction caused by *Campylobacter jejuni*, maintained transepithelial resistance of intestinal cell monolayers, and prevented permeability of intestinal epithelial cells [134]. *L. johnsonii* 6084 mitigated a decrease in gut microorganism diversity and abundance, restored the *Mycobacterium anisopliae* and the Aspergillus phylum abundance in LPS-treated mice, and adjusted the balance of gut microorganisms as a means of enhancing intestinal barrier function [93]. Reports indicate that *L. reuteri* NK33 alleviates gut microbiota imbalance by reducing the number of Proteus bacteria and increasing the number of *Clostridium* bacteria [135]. *L. reuteri* DSM17938 regulated the gut microbiota, increasing bacterial diversity to protect the intestinal barrier [136]. Synthetic bacteria composed of *L. reuteri* and inulin protected the integrity of the intestinal barrier, altered the composition of the intestinal microbiota, and increased the abundance of *Bifidobacterium butyricum* [137]. *L. reuteri* KUB-AC5 could lead to the enrichment of potentially beneficial lactic acid bacteria and inhibit *Proteobacteria*, including nonbeneficial bacteria, to protect the intestinal barrier [138]. *L. reuteri* ZJF036 increased the *Firmicutes* and *Clostridia* and was also found to decrease the *Firmicutes* to *Bacteroides* ratio. The *Lactobacillus* increased, and the *Turicibacter* and *Blautia* decreased to protect the intestinal barrier [139]. *L. reuteri* FYNDL13 could promote the formation of butyric acid, upregulate the transcription of antimicrobial peptide-encoding genes, and prevent hyperimmune reactions around and in the intestine. In addition, it increased the beneficial bacteria abundance (including *Bifidobacteria*, *Akkermania*, *Cyanobacteria*, and *Spirochaeta oscillosum*) and limited the relative abundance of harmful bacteria (*Bacteroides* and *Subelia*) to protect the intestinal barrier [140]. *L. reuteri* CCFM1175 effectively reduced CAP-induced damage to the ileum and colon by increasing the UCG_014 and *Ackermannia* in *Ruminococcaceae* [141]. Thus, *L. salivarius*, *L. johnsonii*, and *L. reuteri* strains improved the intestinal barrier by inhibiting harmful bacteria and increasing beneficial bacteria in the gut.

Thirdly, *L. salivarius*, *L. johnsonii*, and *L. reuteri* strains improve the intestinal barrier by regulating the intestinal immune function. *L. salivarius* 160 upregulated the intestinal IL-6 and TLR2 expression to maintain the intestinal barrier integrity [131]. The addition of *L. salivarius* CML352 decreased the My-D88, IFN-γ, and TLR-4 to improve the intestinal barrier [110,117]. *L. johnsonii* BS15 improved intestinal immunity and enhanced intestinal mucosal immunity by increasing the level of sIgA [142,143]. *L. reuteri* LR1 increased the contents of sIgA, porcine β-defensin 2, and protein 1-5 transcripts of ileum secretory immunoglobulin A to improve intestinal barrier function of weaned pig ileum mucosa [144]. *L. reuteri* f041 could promote intestinal sIgA production and antimicrobial peptide-related gene expression and enhance the mucosal barrier function [145]. In LPS-stimulated mice, *L. reuteri* ZJ617 and ZJ615 modulated intestinal immune responses and metabolism [146]. *L. reuteri* 22 inhibited the Notch signaling pathway, increased the expression of mucin 2, and improved intestinal mucosal immunity [147]. Bovine *L. reuteri* RGW1 increased the levels of TGF-β and IL-10, and its cell-free supernatant (RCS) decreased the serum TNF-α levels [148]. *L. reuteri* D8 enhanced the intestinal mucosal barrier through increasing goblet cells and antimicrobial peptides (AMPs) related gene expression, including *Muc2*, *Lyz1*, and *pBD1* [149]. *L. reuteri* ATCC 55,730 administration induced CD4-positive T lymphocytes in the ileal epithelium of a human intestinal tract [150]. The BBC3-derived ev (lrev) of *L. reuteri* inhibited the Th1- and Th17-mediated inflammatory response by inhibiting NF-κB activity. The activation of macrophages enhanced immunoregulatory cell-mediated splenic lymphocyte immunosuppression and maintained intestinal immune homeostasis [151]. *L. reuteri* TPC32 could enhance intestinal biochemical and physical barrier functions by increasing sIgA expression [152]. In a mouse model of alcoholic leaky gut, *L. reuteri* 3632 modulated gut and immune homeostases by activating aryl hydrocarbon receptors (AhR) [153]. *L. salivarius*, *L. johnsonii*, and *L. reuteri* strains played important roles in maintaining intestinal immune homeostasis, especially *L. reuteri*, which has been reported more frequently.

Furthermore, there are still other mechanisms by which *L. salivarius*, *L. johnsonii*, and *L. reuteri* strains improve the intestinal barrier. *L. salivarius* SMXD51 enhanced intestinal barrier function by increasing transepithelial electrical resistance (TEER) and strengthening the F-actin cytoskeleton [84]. *L. salivarius* 7247 and the *Limosilactobacillus fermentum* 3872 consortium also protected intestinal barrier functions through increasing TEER and inhibiting paracellular permeability in monolayers of human and animal enterocytes [154]. *L. reuteri* NPL-88 could increase the TEER [155], *L. reuteri* I5007 increased the protein expression of intestinal epithelial protein-1 in newborn piglets and maintained the IPEC-J2 cells’ TEER, and its supernatant inhibited an increase in TNF-α and IL-6 expression and a decrease in TJ protein expression induced by LPS [156]. *L. reuteri* 100-23 stimulates the development of regulatory T cells [157]. The specific cell-free fermentation supernatant (CFS) of *L. reuteri* G7 promoted the growth and proliferation of the intestinal epithelial cell line Caco-2 and enhanced the intestinal barrier [158]. In rats and mice, *L. salivarius* LI01 reduces serum endotoxin and bacterial translocation, improves intestinal ultrastructure damage, and maintains the intestinal barrier [159]. *L. reuteri* ZJ617 protected against LPS-induced intestinal barrier dysfunction by enhancing antioxidant activity through the mTOR signaling pathway [160]. Administration of *L. johnsonii* YH1136 ameliorates high-altitude hypoxia-induced intestinal injury by regulating *Staphylococcus* and *Corynebacterium* cooperated with miR-196a-1-3p and miR-3060-3p, respectively [60]. Extracellular vesicles derived from *L. johnsonii* (LJ-EVs) could effectively prevent colitis symptoms because LJ-EVs could be directly absorbed by intestinal epithelial cells, activate the Nrf2/HO-1 antioxidant signaling pathway, reduce endotoxin damage to cells, and thus maintain intestinal barrier homeostasis [161]. Whether there are other mechanisms of action remains to be studied.

Overall, *L. salivarius*, *L. johnsonii*, and *L. reuteri* can ameliorate the intestinal barrier, enhance intestinal immune function, and protect the organism from external influences by increasing intestinal epithelial tight-junction protein expression and mucin production, adjusting and balancing the level of the intestinal microflora, blocking pro-inflammatory cytokines, and so on.

### 3.2. Alleviate Intestinal Inflammatory Response

Systemic inflammation is a natural, protective biological response of the host immune system that fights off harmful foreign pathogens (including bacteria, viruses, and toxins) and helps the body restore health.

*L. salivarius*, *L. johnsonii*, and *L. reuteri* strains reduce intestinal inflammation mainly through regulating inflammatory cytokine expression and secretion. For instance, *L. salivarius* ZLP-4b could improve the intestinal morphology of mice, increase the contents of the anti-inflammatory cytokines IL-4 and IL-10, and decrease the content of the pro-inflammatory factor IL-17A [162]. *L. salivarius* UCC118 intervention attenuated the secretion of IL-8 and the pro-inflammatory response induced by *Salmonella typhimurium* and stimulated the secretion of IL-10 and TNF-α by dendritic cells (DCs) to mediate the response of intestinal pathogens and play an immunomodulatory role [32]. Both *L. salivarius* CCFM 1266 and *L. salivary* UCC118TM alleviated colon inflammation by increasing M2 macrophage polarization and anti-inflammatory IL-10 production [163]. *L. salivarius* CNCM I-4866 reduced the expression of markers of colon injury and inflammation. It could also exert anti-inflammatory effects by reducing the amount of IL-8 produced by TNF-α-stimulated cells and regulating cytokine profiles in peripheral blood mononuclear cells (PBMCs) [17]. Combined treatment with *L. salivarius* Li01 and *Bifidobacterium longum* TC01 more effectively reduced the TNF-α, MCP-1, and M-CSF levels, thereby inhibiting systemic inflammation in rats [115]. The combination of RJGP16 and *L. salivarius* B1 treatment significantly increased the levels of IL-6 and porcine β-defencin (pBD)-2 in the duodenum and ileum [164]. *L. johnsonii* L531 preincubation induced the expression of pro-inflammatory cytokines [123]. *L. johnsonii* N5 inhibited the intestinal TNF-α and IL-6 production, and increased the intestinal Peyer’s patch MHCII and CD103 dendritic cell populations and the number of regulatory T cells, and thus reduced the production of the Th17 population and IL-17a production during colitis to increase the expression of IL-10 to ameliorate colonic inflammation [122]. *L. reuteri* plays a very important role in regulating inflammatory cytokines in vivo and in vitro. *L. reuteri* LM1071 displays potential anti-inflammatory capacity, which is achieved by inhibiting the production of inflammatory mediators such as NO, arachidic acids like PGE1 and PGE2, pro-inflammatory cytokines, and COX proteins [165]. Furthermore, it can increase the expression of inflammation-related genes such as *IL-11*, *BMP4*, *LEFTY2*, and *EET* metabolites production. *L. reuteri* MT180537 upregulated the anti-inflammatory cytokines and resulted in the control of *E. faecalis*-MW051601-induced overexpression of pro-inflammatory cytokines [166]. *L. reuteri* ATG-F3 (F3) and F4 strains exhibited anti-inflammatory effects on RAW264.7 mouse macrophages, and mice orally administered with the F4 strain showed increased ileum IL-10 production [167]. *L. reuteri* F-9-35 reduced the colon tissue pro-inflammatory gene expression [168]. The soluble factor of *L. reuteri* CRL1098 significantly decreased the production of NO, COX-2, Hsp70, TNF-α, and IL-6 in LPS-stimulated macrophages [169]. In children with active distal ulcerative colitis, rectal instillation of *L. reuteri* ATCC 55,730 effectively improved mucosal inflammation through increasing IL-10 levels and decreasing IL-1β, TNF-α, and IL-8 levels in the mucosa [170]. Specific strains of *L. reuteri* L3 and L8 were identified in control mice and obese mice, respectively. *L. reuteri* L8 induced the production of IL-6, IL-12, and TNF-α, while *L. reuteri* L3 induced IL-10 production [171]. *L. reuteri* 5454 efficiently triggered IL-22 secretion and regulatory T-cell induction in dendritic cells [172]. *L. ruminis* and *L. reuteri* LY2-2, through regulating pro-inflammatory cytokines production, alleviated DSS-induced colitis [173]. These results illustrated that the *L. salivarius*, *L. johnsonii*, and *L. reuteri* strains regulated the intestinal inflammatory response by decreasing anti-inflammatory factors and reducing pro-inflammatory factors.

**Figure 2:**
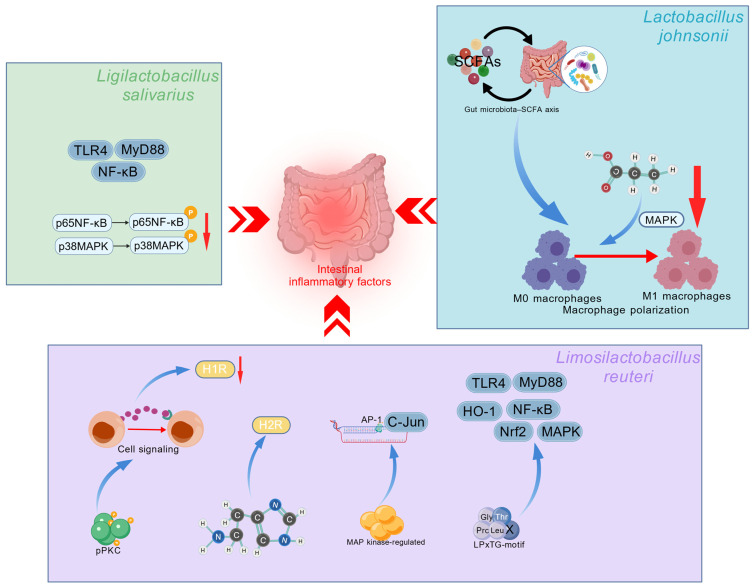
The signaling pathways involved in the *L. salivarius*, *L. johnsonii*, and *L. reuteri* strains regulating inflammatory cytokines. Created with BioGDP.com [114].

The signaling pathways involved in the *L. salivarius*, *L. johnsonii*, and *L. reuteri* strains regulate inflammatory cytokines (Figure 2). Research has shown that *L. salivarius* reduced inflammation-related factors by decreasing the p38 MAPK and p65 NF-κB phosphorylation of IPEC-J2 cells [16]. *L. salivarius* WZ1 inhibited the jejunum inflammatory damage induced by ETEC K88 via the TLR4/NF-κB/MyD88 pathways [15]. *L. johnsonii* inhibited pro-inflammatory cytokines secretion mainly by gut microbiota-derived short-chain fatty acids, suppressing the M1 macrophages polarization [15]. In particular, the increased concentration of propionic acid in the gut inhibited the MAPK signaling pathway activation of macrophages, thereby reducing the polarization of M1 macrophages [174,175]. *L. reuteri* DSM 8533 inhibited TNF-α and IL-1β production by regulating the ERK-JNK-related MAPK signaling cascade through LPxTG-motif surface protein [176]. *L. reuteri* GroEL and LrPGN seemed to inhibit inflammation by the activation of a noncanonical TLR4 pathway [85,177]. *L. reuteri* 17938 exerted its anti-inflammatory effect through the TLR2 pathway [178]. *L. reuteri* 6475 attenuated pPKC-mediated mammalian cell signaling to inhibit the pro-inflammatory response mediated by H1R in the gut [179]. In lipopolysaccharide-activated monocytes and primary monocytes derived from children with Crohn’s disease, the probiotic *L. reuteri* strain ATCC PTA 6475 inhibited TNF transcription via suppressing MAP-regulated c-Jun and activating transcription factor AP-1 [180]. *L. reuteri* LR1 could activate the MLCK signaling pathway of IPEC-J2 cells to inhibit ETEC k88 challenge [181]. *L. reuteri* SH 23-derived LPxTG-motif surface protein had the function to alleviate inflammatory diseases through the NF-κB pathway [182]. *L. reuteri* that carries the amino acid decarboxylase gene converted L-histidine into histamine in the intestinal lumen, activating H2R, which ultimately inhibits acute inflammation in the mouse colon [183]. *L. reuteri* RE225 reduces mice inflammation by inhibiting the TLR4/MyD88/NF-κB and Nrf2/HO-1pathways [184]. These findings improve our understanding and knowledge of how *L. salivarius*, *L. johnsonii*, and *L. reuteri* reduce intestinal inflammation and provide new insights for human disease and animal treatment target identification.

## 4. Relieving the Progression of Diseases

In animal models and clinical trials, it has been found that *L. salivarius* has been used to prevent and treat multiple human chronic diseases, including asthma, cancer, atopic dermatitis, and halitosis, and has also been used for infection prevention or treatment [11]. For example, *L. salivarius* LI01 and *L. salivarius* LI02 have been demonstrated to prevent acute liver failure in rats [185]. *L. salivarius* WB21 inhibited the quantity of oral periodontopathic bacteria, such as *Porphyromonas gingivalis*, *P. intermedia*, *Tannerella forsythensis*, and *Fusobacterium nucleatum* [186]. *L. salivarius* LI01 plays a protective role against thioacetamide (TAA)-induced acute liver injury and hyperammonemia in mice [187]. A 14-day oral administration of *L. salivarius* K12 was conducted on hospitalized COVID-19 patients and confirmed the hypothesis that oral microbiota directly participates in the lung microbiota establishment, significantly reducing the mortality rate associated with COVID-19 infection [188]. Nadja Larsen et al. found that *L. salivarius* Ls-33 modified the fecal microbiota in obese adolescents in a way not related to metabolic syndrome [189].

The patient underwent surgical resection of <1 cm, removing all visible lesions in the past 21 days, and received encapsulated freeze-dried *L. johnsonii* LA1 (2 × 10^9^ CFU) twice daily, resulting in a reduction of endoscopic recurrence from 64% to 49% [190]. *L. johnsonii* N6.2 reduced the oxidative response protein expression (i.e., Gpx1, GR, and Cat) in the intestinal mucosa and inhibited the onset of type1 diabetes in rats [191]. Xin et al. reported that *L. johnsonii* BS15 inhibited non-alcoholic fatty liver disease (NAFLD)-associated insulin resistance in mice, and reduced the gene expression of acetyl-CoA carboxylase 1 (ACC1), fatty acid synthase (FASN), and peroxisome proliferator-activated receptor gamma (PPARγ); it also increased the expression of fasting-induced adipokines in the liver of obese mice [192]. *L. johnsonii* BS15 alleviated abnormal mitochondrial by decreasing uncoupling protein 2 and increasing cytochrome C levels in diabetes [193]. Yin et al. found that heat-killed *L. johnsonii* (HKLJ) upregulated the intestinal lysozyme expression in alcohol-related liver disease, and enhanced intestinal bacteria-mediated immunoregulatory substances production [194]. This activation leads to the NOD2-IL-23-IL-22 innate immune axis activation, and elevated IL-22 upregulates the synthesis of antimicrobial peptides to maintain intestinal homeostasis. Additionally, HKLJ also activates the liver signal transducer and activator of the transcription 3 (STAT3) pathway and promotes liver damage repair [195].

A review summarized that *L. reuteri* was used as a probiotic for the treatment of functional abdominal pain, diarrhea, constipation, *H. pylori* infection, IBD, diverticulitis, colorectal cancer, and liver disease [196]. *L. reuteri also* contributes to alleviating depression, which has been confirmed in studies involving both mice and humans [48,197]. Samiraninezhad et al. found that a probiotic nanomedicine containing *L. reuteri* treatment with recurrent aphthous stomatitis for one week significantly increased the lesion size and reduced the severity of pain compared to the control group [198]. In the first use of *L. reuteri* selenium nanoparticles for the treatment of ulcerative colitis in mice, the prepared SeNPs effectively alleviated symptoms such as diarrhea, weight loss, bloody stools, and colon shortening [199]. The decrease in the number of *L. reuteri* in the human body has been positively correlated with the increase in the incidence of inflammatory diseases over the same period. Direct supplementation of *L.reuteri* or the regulation of its content through prebiotics is a promising method for the prevention and/or treatment of diseases.

Taken together, *L. salivarius*, *L. johnsonii*, and *L. reuteri* strains play a significant and important role in promoting human health. They could provide certain protective effects against various health issues in humans, such as diarrhea, constipation, colorectal cancer, and liver diseases. Additionally, they have been found to have the potential to suppress the onset of diabetes. However, their role in human clinical research is still limited (Table 2). Therefore, developing these three probiotics as therapeutic agents for human diseases has broad research prospects.

## 5. Summary and Outlook

*L. salivarius*, *L. johnsonii*, and *L. reuteri* strains can regulate the intestinal barrier and enhance intestinal immune function through various mechanisms, such as increasing the expression of tight-junction proteins and mucin secretion in intestinal epithelial cells, adjusting and balancing intestinal microbiota, and blocking pro-inflammatory cytokine production. They have been shown to reduce intestinal inflammation in multiple animal models and provide protective effects against various health issues in humans, such as diarrhea, constipation, colorectal cancer, and liver diseases.

However, the detailed mechanism of some strains remains unclear, and new and powerful strains still need to be isolated and identified. The probiotic properties depend on their genome, and new microbiological strategies, such as mutational breeding of microbial strains, can better enhance the probiotic properties of strains lacking genetic manipulation systems. In addition, fecal metagenomic sequencing, such as exfoliome sequencing (Foli-seq), was used to profile fecal exfoliated eukaryotic messenger RNAs (feRNAs) originating from the upper and lower gastrointestinal regions, and metabolomics studies have revealed the impact of the gut microbiota on health and disease. Microorganisms have unique and efficient biotransformation capabilities and can produce a variety of metabolites. With the development of metabolomics, more metabolites of *L. salivarius*, *L. johnsonii*, and *L. reuteri* will be identified and produced by synthetic biology technology in the future, and both probiotics and their products have broad application prospects in the fields of biology, medicine, and food and animal feed production.

## Figures and Tables

**Table 1:** Resistance of *L. salivarius*, *L. johnsonii*, and *L. reuteri* to pathogens in different experiments ^1^.

Pathogens	Bacterial Strain	Source	Mechanism	References
*Escherichia coli* (*E. coli*)	*L. salivarius* FFIG35 and FFIG58	Swine intestinal	IFN-β, IFN-λ, and antiviral factors ↑. Regulating the immune response of PIE cells involves negative regulators of the TLR signaling.	[14]
	*L. salivarius* WZ1	Calf	TNF-α, IL-1β, and IL-6 ↓. Regulating the TLR4/NF-κB/MyD88 inflammatory pathway and gut microbiota.	[15]
	*L. salivarius*	Feces of piglets	Attenuating phosphorylation of p38 MAPK and blocking the NF-κB signaling pathways enhances the integrity of IPEC-J2 cells.	[16]
	*L. salivarius* CNCM I-4866	Rumen of grazing lamb	Lactic acid production.	[17]
	*L. salivarius*	Chicken droppings	Improves intestinal flora composition, reduces lung inflammatory damage, and enhances host defense.	[18]
	*L. salivarius*	Swine	Increased fecal *Lactobacillus* populations ↑ and improved intestinal morphology.	[19]
	*L. johnsonii* LJ1	Tibetan yak	Regulates intestinal flora and reduces diarrhea symptoms.	[20]
	*L. johnsonii* L531	Colon of weaned piglet	Limiting the activity of the NLRP3 inflammasome induces autophagy by promoting ATG5/ATG16L1-mediated autophagy.	[21]
	*L. reuteri* HCM2	Healthy piglet	Regulates intestinal flora in mice.	[22]
	*L. reuteri* (E, KO5, CCM 3625, ATCC 55730)	Lamb, goat stomach, Paste-rennet, human	Production of organic acids, ethanol, and Reuterin.	[23]
	*L. reuteri* ATCC PTA 6475	Human	Inhibited colonization.	[24]
	*L. reuteri*	Human	Antibacterial factor synergistic with Reuterin.	[25]
	*L. reuteri* WHH1689	Chinese traditional highland barley wine	Inhibitory activity against *Escherichia coli*, *Shigella flexneri*, *Salmonella paratyphi β*, and *Staphylococcus aureus*.	[26]
	*L. reuteri* JCM 1081	Chicken intestine	Inhibits bacterial adhesion.	[27]
	*L. reuteri* TMW1.656 and LTH5794	Sour dough, human intestines	Inhibits the colonization level.	[28]
*Salmonella*	*L. salivarius* 7274	The gut and reproductive tract of healthy women	Lactic acid (LA) and bacteriocins.	[29]
	*L. salivarius*	Chicken droppings	Specific antibodies, IFN-γ, and lymphocytes ↑ degrade AFB1.	[30]
	*L. salivarius* CNCM I-4866	Rumen of grazing lamb	LA.	[17]
	*L. salivarius* L61 and L55	Chicken manure	Upregulated heterophil phagocytosis and phagocytic index (PI).	[31]
	*L. salivarius* UCC118	Human gut	IL-8 ↓, stimulates dendritic cells (DC) to secrete IL-10 and TNF-α ↑.	[32]
	*L. salivarius* CECT 5713	Human breast milk	Inhibits adhesion and increases the expression of intestinal mucin.	[33]
	*L. salivarius* CTC2197	Chick gastrointestinal tract	Reduces bacterial colonization.	[34]
	*L. johnsonii*	Chicken	Competitive exclusion and reduced colonization.	[35]
	*L. salivarius* TUCO-L2	The milk of South American camel	TNF-α ↓, IFN-γ, and IL-10 ↑.	[36]
	*L. johnsonii* NCC 533	Human gut	Hydrogen peroxide is produced and effectively kills the model pathogen Salmonella enteritidis serotype *Salmonella Typhimurium* SL1344.	[37]
	*L. reuteri*		Reuterin.	[38]
*Salmonella*	*L. johnsonii* L531	Colon contents of weaned piglets	NOD activated ↓ regulates endoplasmic network stress and promotes autophagy degradation. Regulates T-cell response to maintain intestinal homeostasis Clears damaged mitochondria and regulates the NF-κB-SQSTM1 mitochondrial autophagy signaling pathway. Inhibits colonization and reduces SCFAs consumption. Iron homeostasis and oxidative stress are regulated through the IRP2 pathway.	[39,40,41,42,43]
	*L. reuteri* ATCC 55730 and L22	Human	Reuterin.	[44]
	*L. reuteri* WHH1689	Chinese traditional highland barley wine	Inhibitory activity against *Escherichia coli*, *Shigella flexneri*, *Salmonella paratyphi β*, and *Staphylococcus aureus*.	[26]
	*L. reuteri*	Human	Antibacterial factor synergistic with Reuterin.	[25]
	*L. reuteri* ATCC 53608	Swine	Activates the PI3K/AKT pathway.	[45]
	*L. reuteri* Lb11	Chicken intestinal tract	AcrAB-TolC efflux pump genes, outer membrane protein genes, and antibiotic resistance genes ↓.	[46]
	*L. reuteri* S5	Healthy broiler	Inhibits growth and adhesion, inhibits virulence and cell membrane integrity gene expression, inhibits biofilm formation, destroys bacterial structure, and inhibits protein synthesis.	[47]
	*L. reuteri* ATCC 55730	Human	Activates macrophages, regulating NO.	[48]
	*L. reuteri* PFS4	Poultry intestine	Inhibited biofilm formation; cell-free supernatant (CFS) reduced growth and adhesion.	[49]
	*L. reuteri* JCM 1081	Chicken intestine	Inhibits bacterial adhesion.	[27]
	*L. reuteri* KUB-AC5	Chicken intestines	Combination with *U. rigida*, exhibited synergistic activity.	[50]
	*L. salivarius* XP132	Pheasant	Prevents transmission.	[51]
*Staphylococcus*	*L. salivarius* CNCM I-4866	Rumen of grazing lamb	Lactic acid production.	[17]
	*L. salivarius* AR809	Healthy population pharynx	Regulates TLR/PI3K/Akt/mTOR signaling pathway-related autophagy and TLR/PI3K/Akt/IκB/NF-κB pathway activity.	[52]
	*L. salivarius* CICC 23174	Hen droppings	Inhibits the adhesion of Staphylococcus aureus.	[53]
	*L. johnsonii* LJO02 cell-free supernatant	Healthy human gut	Reduces pathogenicity and promotes wound healing.	[54]
	*L. salivarius*	Oral mucosa in healthy children	Five secreted proteins, including lysm’s peptidoglycan-binding protein and a protein peptidase; regulates PH.	[55]
	*L. reuteri* ATCC 55730	Human breast milk	Competitive rejection inhibits adhesion to keratinocytes.	[56]
	*L. reuteri* strains	Human, rat	Inhibits growth.	[57]
	*L. reuteri* WHH1689	Chinese traditional highland barley wine	Inhibitory activity against *Escherichia coli*, *Shigella flexneri*, *Salmonella paratyphi β*, and *Staphylococcus aureus*.	[26]
*Clostridium perfringens*	*L. johnsonii*	Chicken	Competitive exclusion and inhibitory colonization.	[35]
	*L. johnsonii* FI9785	Poultry	Inhibition of colonization and persistence of *Bacillus perfringens*.	[58]
Human Immunodeficiency Virus Type 1 (HIV-1)	*L. salivarius* CECT 5713	Human breast milk	Stimulates immature dendritic cells to mature.	[59]
Endogenous pathogenic bacteria	*L. johnsonii* YH1136	High-altitude Tibetan girl	Regulates intestinal flora, increases the abundance of lactic acid bacteria, and reduces the abundance of pathogenic bacteria.	[60]
Uremic toxins	*L. salivarius* JCM1231	Human saliva	Increased cell activity and apoptosis, IL-6, and TNF-α ↑.	[61]
Mycotoxins	*L. salivarius* SMXD51	The cecum of poultry	Changes the bacterial genus of poultry intestinal microbiota to limit the Influence of campylobacter on *Anaerotruncus sp.* decrease and *Subdoligranulum sp.* increase.	[62]
*Helicobacter pylori* (*H. pylori*)	*L. salivarius* subsp. *salicinius* AP-32	Taiwanese	Reduces the *H. pylori* load in the gastric mucosa, and reduces inflammatory chemokine expression and lymphocyte infiltration.	[63]
	*L. johnsonii* MH-68	Indigestion of the stomach	NF-κB ↓.	[64]
	*L. salivarius* B37 and B60			
	*L. johnsonii* No. 1088 (HK-LJ88)	The stomach juices of healthy Japanese	Deformations of *H. pylori* (e.g., disappearance of spiral, bending of cell body, coccoid formation, degradations, etc.).	[65]
	*L. johnsonii* La1	Human feces	Peptide extracts from cultures of *Lactobacilli* inhibit colonization and inflammation.	[66]
	*L. johnsonii* 1088	Human gastric juice	Reduces *Helicobacter pylori* infection.	[67]
	*L. reuteri* 2892	Camel milk	Reduces dead cells and apoptotic cells.	[68]
	*L. reuteri* SD2112	Human breast milk	Inhibits urease activity and reduces pathogen density.	[69]
	*L. reuteri* 17938	Human breast milk	Secretes Reuterin and Reutericycline.	[70]
Respiratory syncytial virus (RSV)	*L. johnsonii*	Mouse cecum	Immunomodulatory metabolites mediate airway mucosal protection and alter immune function.	[71]
*Campylobacter jejuni* (*C. jejuni*)	*L. salivarius* NRRL B-30514	Chick cecum	Bacteriocin.	[72]
	*L. johnsonii* FI9785	Poultry	Reduced colonization of cecal contents	[73]
	*L. salivarius*, *L. johnsonii*, *L. reuteri*	Chicken	Growth inhibition, quorum-sensing molecular autoinducer-2 (AI-2) ↓.	[74]
	*L. salivarius* UO.C249		Production of Extracellular vesicles (EVs) and bacteriocins.	[75]
	*L. salivarius*	Chicken droppings	Improves intestinal flora composition, reduces lung inflammatory damage, and enhances host defense.	[18]
*Mycoplasma gallisepticum*	*L. salivarius* FFIG35 and FFIG58	Pig intestine	IFN-β, IFN-λ, and antiviral factors ↑. Regulated the immune response of PIE cells; negative regulators of the TLR signaling. Reduces rotavirus replication in PIE cells.	[14,76]
Rotavirus	*L. reuteri* Probio-16	Pig manure	Cell-free supernatant (CFS) inhibits enteric bacterial pathogens and porcine rotavirus.	[77]
	*L. reuteri*	Human	Rotavirus-specific antibodies ↑.	[78]
	*L. salivarius*	Chicken	Specific antibodies, IFN-γ, and lymphocytes ↑.	[79]
Infectious bursal disease virus (IBDV)	*L. salivarius* BP121	Infant feces	Reduces inflammation and oxidative stress, and regulates the intestinal environment.	[80]
*Porphyromonas gingivalis*	*L. reuteri*	Breast milk	Reuterin.	[81]
*Enterococcus faecalis*	*L. reuteri*	Breast milk	Reuterin.	[81]
*Propionibacterium acnes* *P. acnes*	*L. salivarius* LS03		Actives bacteriocin against the proliferation of *Propionibacterium acnes* and *Staphylococcus epidermidis.*	[82]
	*L. reuteri* strains (KCTC 3594 and KCTC 3678) *L. reuteri* KCTC 3679	Human, rat	Inhibits growth.	[57]
*Pseudomonas aeruginosa*	*L. salivarius*	Healthy oral cavity for adults	Pro-inflammatory cytokines and antibiotic membranes ↑.	[83]
	*L. salivarius* SMXD51	The cecum of Tunisian poultry	Increases TEER and enhanced F-actin cytoskeleton to enhance intestinal barrier function.	[84]
	*L. reuteri* (E, KO5, CCM 3625 and ATCC 55730)	Lamb, goat stomach, Paste-rennet, human	Production of organic acids, ethanol, and Reuterin.	[23]
*Aspergillus hydrophilus*	*L. salivarius* ATCC 11741	Human saliva	Activity of lysozyme (LYZ), phenoloxidase (PO), nitrogen synthase (NOs), and alkaline phosphatase (AKP) ↑.	[85,86]
*Cronobacter sakazakii*	*L. salivarius* YL20	Breast milk	ZO-1, Occludin ↑, reversed the decrease of transepithelial resistance (TEER) and the increase of permeability of Caco-2 monolayer cells.	[87]
Aflatoxin B1 (AFB1)	*L. salivarius*	Chicken droppings	Specific antibodies, IFN-γ, lymphocytes ↑ degrade AFB1.	[30]
	*L. reuteri*		Combination.	[88]
Porcine epidemic diarrhea virus (PEDV)	*L. salivarius* JCM		GRP78 (glucose regulatory protein 78) ↓. FAK/PI3K/Akt signaling pathway ↑.	[89]
	*L. johnsonii*-COE	Pig intestinal mucus	The monocyte-derived MoDC is stimulated to maturity and triggers a cellular immune response, inducing the increase of serum IgG, IgA, and IgM, and mucosal SIgA secretion of pregnant sows.	[90]
	*L. reuteri* C8	Pig manure	Prophylactic, therapeutic, competitive, and direct-inhibitory actions.	[91]
*Proteobacteria* and *Spirochaetes*	*L. salivarius* zlp-4b	Pig	Increases the relative abundance of lactic acid bacteria and reduces the relative abundance of opportunistic pathogens.	[92]
Sepsis	*L. johnsonii* 6084	Pig rectum	Improves gut microbial diversity.	[93]
	*L.reuteri* WXD171	Dairy product	Induces the mucosal response of intestinal-associated lymphoid tissue.	[94]
*Candida* *glabrata*	*L. johnsonii*	Mouse feces	Reduces inflammatory parameters, reduces *E. coli* and *Enterococcus faecalis* populations, and eliminates Candida glabra from the gut.	[95]
	*L. johnsonii*		Promotes the elimination of *C. glabrata* from the gut via chitinase-like and mannosidase-like activities.	[96]
	*L. johnsonii*	Oral cavity of mice infected with *C. albicans*	Inhibits the growth of Candida and inhibits the growth of potentially synergistic bacteria (such as enterococcus) to inhibit the candida virulence.	[97]
	*L. johnsonii* MT4	The mouth of a C57BL/6 mouse	The floating growth and biofilm formation of *Candida albicans* were inhibited by pH-dependent and pH-independent antagonisms.	[98]
	*L. reuteri* RC-14	Woman’s vagina	Stagnant growth leads to cell death.	[99]
Subclinical Necrotic Enteritis (SNE)	*L. johnsonii* BS15	Grassland homemade yogurt	Improves lipid metabolism and intestinal flora.	[100]
*Fusobacterium nucleatum*	*L. reuteri*	Breast milk	Reuterin.	[81]
*Streptococcus mutans*	*L. salivarius* K35 and K43	Human saliva	Inhibits the growth and expression activity of *Streptococcus mutans* virulence genes to reduce the formation of its biofilm.	[101]
	*L. reuteri* (KCTC 3594 and KCTC 3678) and *L. reuteri* KCTC 3679	Human and rat	Inhibits biofilm formation, production of organic acids, hydrogen peroxide, and a bacteriocin-like compound.	[102]
	*L. reuteri*		Interfered with *S. mutans* biofilm formation in vitro, and that the antimicrobial activity against *S. mutans* was pH-dependent.	[103]
White spot syndrome (WSSV)	*L. johnsonii* KD1	The gut of a European bass	The bacteriotin helveticin-J homolog can block the VP28-PmRab7 interaction and interrupt WSSV infection.	[104]
Coxsackievirus type A (CA) strain 6 (CA6), CA16 and EV71	*L. reuteri* Protectis	Breast milk	Physical interaction.	[105]
*Fusarium verticillioides* 97 L	*L. reuteri* LR-92		Cell-free supernatant (CFS) bactericidal action and inhibitory activity.	[106]
*Candida. albicans*	*L. reuteri* RC-14	Healthy female vagina	Inhibits metabolic activity.	[107]
	*L. reuteri* RC-14	Healthy female vagina	Cell-free supernatant (CFS) may upregulate IL-8 and IP-10 secretion by VK2/E6E7 cells.	[108]
	*L. reuteri*	Breast milk	Reuterin.	[81]
*Shigella sonne*	*L. reuteri*	human	None	[25]
*Shigella flexneri*	*L. reuteri* WHH1689	Chinese traditional highland barley wine	Inhibitory activity against *Escherichia coli*, *Shigella flexneri*, *Salmonella paratyphi β*, and *Staphylococcus aureus*.	[26]
*Listeria monocytogenes*	*L. reuteri* INIA P579		Reuterin	[109]
	*L. salivarius* C2-1	Broiler intestine	Bacteriocin C2-1 affects cell membrane permeability and integrity, leading to the leakage of intracellular substances.	[110]
Porcine circovirus type 2 (PCV2)	*L. reuteri* L26		Stimulates the intestinal immune response.	[111]
Influenza A/PR8, LD50	*L. reuteri* KBL346	Infant feces	Alleviates disease severity and improves histopathological changes.	[112]
*Enterococcus faecalis*	*L. reuteri* JCM 1081	Chicken intestine	Inhibit bacterial adhesion.	[27]

^1^ Herein, ↑ represent upregulation and ↓ represent downregulation.

**Table 2:** Main effect of *L. salivarius*, *L. johnsonii*, and *L. reuteri* on human disease in clinical trials.

Strains	Main Effect on Human Disease in Clinical Trials	References
*L. salivarius* WB21, WB24,	Periodontal health and halitosis	[11,200,201]
TI 2711		
*L. salivarius* LS01	Atopic dermatitis	[202]
*L. salivarius* Ls-33	Obesity	[189]
*L. salivarius* SGL03	COVID-19 infections	[203]
*L. salivarius* AR809	Pharyngitis	[52]
*L. johnsonii* NCC 533	Gastrointestinal disease	[10]
*L. johnsonii* MH-68	Type 1 diabetes	[204]
*L. johnsonii* BS15	Metabolic diseases	[204]
*L. johnsonii* La1	Colorectal cancer	[205]
*L. johnsonii* GDMCC1.730	Chronic kidney disease	[206]
*L. johnsonii* No. 1088	Temporal heartburn symptoms	[207]
*L. reuteri* DSM 17938	Colic	[208]
*L. reuteri* JBD301	Obesity	[5]
*L. reuteri* ATCC 55730	Inflammatory bowel disease	[209]
*L. reuteri* TSR332	Metabolic-associated fatty liver disease	[210]
*L. reuteri* CCFM1040	Allergic rhinitis and asthma	[211]
*L. reuteri* V3401	Metabolic syndrome	[212]
*L. reuteri* FN041	Atopic dermatitis	[1]

## Data Availability

No new data were created or analyzed in this study. Data sharing is not applicable to this article.

## Abbreviations

GIT	Gastrointestinal tract
SE	*Salmonella enteritidis*
TU	Translocable units
lncRNA	Long noncoding RNA
WGS	Whole-genome sequencing
SalB	Class II bacterial salivary protein B
WSSV	White spot syndrome virus
BSH	Bile salt hydrolase
EPS	Extracellular polysaccharide
MAGs	Metagenome-assembled genomes
CRISPR–Cas	CRISPR loci together with Cas genes
ETEC	*Escherichia coli*
TNF-α	Tumor necrosis factor-α
IFN-γ	Interferon-γ
IL	Interleukin
NF-κB	Nuclear factor kappa-B
IκB	Inhibitor of NF-κB
TLR	Toll-like receptor
LYZ	Activity of lysozyme
PO	Phenoloxidase
NOs	Nitrogen synthase
AKP	Alkaline phosphatase
NOD	Nucleotide oligomerization domain
PI	Phagocytic index
IFITM3	Interferon-Induced Transmembrane Protein 3
PI3K/Akt	The phosphatidylinositol 3-kinase/Akt
AI-2	Autoinducer-2
IRF7	Interferon regulatory factor 7
OAS	2′,5′-oligoadenylate synthase
ATG16L	Atg16-like
NLRP3	NOD-like receptor family pyrin domain containing 3
AFB1	Aflatoxin B1
PEDV	Porcine epidemic diarrhea virus
MG	*Mycoplasma gallisepticum*
IBDV	Infectious bursal disease virus
RSV	Respiratory syncytial virus
OS	Oxidative stress
ROS	Reactive oxygen species
SCFAs	Short-chain fatty acids
MDA	Malondialdehyde
SOD	Superoxide dismutase
GSH-Px	Glutathione peroxidase
CAT	Catalase
Nrf2	Nuclear factor-erythroid 2-related factor 2
HO-1	Haem oxygenase-1
ALT	Alanine aminotransferase
POD	Peroxidase
LPS	Lipopolysaccharides
Ig	Immunoglobulins
SNE	Subclinical necrotic enteritis
Lgr5	Leucine-rich repeat-containing G protein-coupled receptor 5
Zo-1	Zonula occludens-1
TEER	Transepithelial resistance
JAM-2	Junctional adhesion molecule-2
VH	Villus height
DC	Dendritic cells
PBMC	Peripheral blood mononuclear cells
pBD-2	Porcine β-defencin-2
CHOP	C/EBP homologous protein
ATF6A	Activating transcription factor 6α
2DG	2-deoxy-D-glucose
PYY	Peptide YY
IPEC-1	Intestinal porcine epithelial cell line
TAA	Thioacetamide
LBP	Lipopolysaccharide-binding protein
BDNF	Brain-derived neurotrophic factor
AgNPs	Silver nanoparticles
PEG	Polyethylene glycol
DSS	Dextran sulfate sodium
MLN	Mesenteric lymph nodes
NO	Nitric oxide
NAFLD	Non-alcoholic fatty liver disease
ACC1	Acetyl-CoA carboxylase 1
STAT3	Signal transducer and activator of transcription 3
PPARγ	Peroxisome proliferator-activated receptor γ
